# Adsorption Phenomenon of VOCs Released from the Fiber-Reinforced Plastic Production onto Carbonaceous Surface

**DOI:** 10.3390/polym15071640

**Published:** 2023-03-25

**Authors:** Joon Hyuk Lee, Eunkyung Jeon, Jung-kun Song, Yujin Son, Jaeho Choi, Seongjun Khim, Minju Kim, Ki-Ho Nam

**Affiliations:** 1 Agency for Defense Development, Yuseong P.O. Box 35, Daejeon 34186, Republic of Korea; 2 Department of Textile System Engineering, Kyungpook National University, Daegu 41566, Republic of Korea

**Keywords:** volatile organic compound, activated carbon, adsorption, isotherm, kinetics

## Abstract

The manufacturing of fiber-reinforced plastics has been linked to the discharge of volatile organic compounds (VOCs), particularly toluene and benzene, which have been identified as posing substantial risks to human health and the environment. To counteract this issue, activated carbons have been suggested as a means of reducing VOC emissions through adsorption. The objective of this study was to investigate the adsorption characteristics of toluene and benzene onto activated carbons produced from coal (AC) and coconut shells (CAC). The study was carried out in an aqueous medium. The findings revealed that the AC sample with higher surface characteristics exhibited a higher adsorption capacity (toluene: 196.0784 mg g^−1^ and benzene: 181.8182 mg g^−1^) in comparison to the CAC sample (toluene: 135.1351 mg g^−1^ and benzene: 116.2791 mg g^−1^). The superior adsorption performance of AC on both VOCs can be attributed to its higher surface characteristics. The Langmuir model was found to be more appropriate than the Freundlich model, as indicated by the higher coefficient of determination (R^2^) value of the Langmuir isotherm (avg. R^2^ = 0.9669) compared to that of the Freundlich isotherm (avg. R^2^ = 0.9654), suggesting the use of a monolayer adsorption mechanism. The adsorption kinetics of the samples were analyzed using the pseudo-first-order and pseudo-second-order models, and the former was found to be more fitting, indicating that the rate of adsorption is directly proportional to the concentration difference between the solution and the sample surface. The adsorption process was found to be spontaneous and favorable based on the positive value of ΔG_ads. Furthermore, the adsorption process was endothermic and disordered, as indicated by the positive values of ΔH_ads and ΔS_ads. The regeneration efficiency of all the samples was secured more than 95% upon the fifth cycle.

## 1. Introduction

The production of fiber-reinforced plastics has long been plagued by the release of volatile organic compounds (VOCs), presenting a grave concern that demands immediate attention from the scientific community [1,2]. Through tireless research and experimentation, a myriad of innovative methodologies has been developed to combat the pernicious effects of VOCs [3,4,5]. One such methodology, which has gained widespread acceptance among experts in the field, is the implementation of advanced sensing and monitoring systems. These sophisticated systems are capable of detecting and measuring VOC concentrations in real-time, thus enabling the identification of emission sources and the implementation of targeted control measures to reduce the release of VOCs into the environment. Numerous types of cutting-edge sensors are utilized for VOC monitoring, including photoionization detectors, metal oxide sensors, and gas chromatography. Another popular technique for VOC remediation involves catalytic degradation, which utilizes specialized catalysts to convert harmful VOCs into benign substances such as water and carbon dioxide. Thermal oxidation, which employs high temperatures to break down VOCs into harmless products, is also a widely used method for VOC pollution control. A variety of thermal oxidizers, such as regenerative thermal oxidizers, catalytic oxidizers, and thermal recuperative oxidizers, are employed for this purpose. Finally, adsorption technology, which utilizes potent adsorbents such as activated carbon and zeolites to eliminate VOCs from the air and water, has proven to be an effective and reliable VOC remediation solution.

Among VOCs, toluene and benzene require sufficient treatment among volatile organic compounds due to their toxic nature and potential health hazards [6,7]. Toluene, a colorless and flammable liquid, is utilized as a solvent and starting material for the synthesis of other chemicals. Despite its mild toxicity, it can induce respiratory and nervous system problems if inhaled in high concentrations. Benzene, on the other hand, is a colorless and flammable liquid with a sweet, pleasant odor. It is extensively utilized as a solvent and starting material for the synthesis of other chemicals, with its widespread use leading to its classification as a carcinogenic substance. Prolonged exposure to high levels of benzene can result in serious health issues, such as leukemia.

Considering these concerns, several strategies have been proposed to mitigate the release of toluene and benzene from FRP production. One such approach is the utilization of activated carbons [8,9,10,11]. Activated carbons are porous materials that are produced by carbonizing organic matter, such as coconut shells, coal, or wood, followed by activation in an oxidizing environment. This process results in a highly porous structure with a large surface area, which provides ample opportunities for chemical interactions. Activated carbons exhibit high adsorption capacities due to the presence of various surface functional groups, such as hydroxyl, carboxyl, and carbonyl groups. These groups interact with VOCs through physical adsorption and/or chemical adsorption [12]. Physical adsorption is a result of the van der Waals forces that occur between the surface of the activated carbon and the VOC molecule. Chemical adsorption involves the reaction between the surface functional groups and the VOC molecule, leading to the formation of a chemical bond. The high surface area of activated carbons and the presence of surface functional groups result in a large adsorption capacity for VOCs, causing activated carbons to be a highly effective solution for removing VOCs from the air.

The primary objective of our study was to delve into the adsorption phenomenon of toluene and benzene onto activated carbons. For the purpose of achieving a comprehensive comparison, we utilized two activated carbons as samples: coal-based and coconut-based activated carbons. There exists a plethora of early works exploring coal-based or coconut-based activated carbons. To the best of our knowledge, none of them have explicitly focused on studying both in the same environment. By subjecting both activated carbons to the same adsorbing environment, our research endeavors to facilitate a more effective comparison of carbonaceous adsorbents. This aspect is crucial in filling the knowledge gap, as it allows for a comparative understanding of behavior and the performance of both materials under identical conditions. To analyze the adsorption trend of the samples, the Langmuir and Freundlich isotherms were employed for the experimental calculations. These isotherms are commonly used in the field of adsorption to describe the equilibrium between the adsorbate and the adsorbent surface. The results obtained from the kinetics were further analyzed to understand the underlying mechanisms that govern the adsorption of toluene and benzene onto activated carbons. By synthesizing and building upon the knowledge provided by earlier works, our work offers a valuable perspective by presenting the direct comparison of two activated carbons. This targeted approach allows for a more precise understanding of the adsorption process, with findings that can be directly applied to address pressing environmental concerns.

## 2. Methodology

The preparation of coal-based activated carbon (AC) commences by utilizing pitch as the initial building block. The primary step in the production of AC revolves around the removal of impurities and an increase in carbon contents [13]. The oxidation process necessitates heating the raw pitch in the presence of steam. Here, oxidation was performed under steam conditions at a temperature of 900 °C for a duration of 2 h. The carbonization stage involves heating the oxidized material while prohibiting any interaction with oxygen or air. The main objective of this phase is to transform the carbon-rich material into a porous structure that can adsorb a wide array of impurities [14]. The process of carbonization facilitates the thermal decomposition of the material, which leads to the breakdown of complex organic compounds into simpler carbon molecules. Subsequently, the activation follows carbonization, which entails treating the carbonized material with an activation agent such as CO_2_ or steam [15]. The principal purpose of this stage is to boost the porosity and improve the adsorption capacity. During activation, the sample undergoes further expansion of its micropores, culminating in a highly porous structure. Furthermore, the activation process introduces additional functional groups on the surface of the material, enhancing its adsorption capacity for specific impurities. Carbonization was carried out in an N_2_ environment for a duration of 2 h within a temperature range of 800–900 °C, followed by activation for another hour in a steam atmosphere. The resulting samples were labeled as AC800, AC850, and AC900, indicating their respective carbonization and activation temperatures. Conversely, the production of coconut-based activated carbons was performed following the established protocols in the literature [16]. Briefly, coconut shells, screened to 10–20 mesh and uncontaminated, were carbonized in an N_2_ environment within a temperature range from room temperature to 500 °C. The activation was conducted under steam conditions at a temperature of 900 °C for a duration of 2 h. The resulting sample underwent full neutralization and dehydration before analysis. The coconut-based activated carbons were designated as the CAC sample.

To observe the physical characteristics of the samples, the Brunauer–Emmett–Teller (BET) and Barrett–Joyner–Halenda (BJH) methods were used (ASAP 2020, Micromeritics) [17]. To gain insights into the chemical characteristics of the samples, a CHNS-O analyzer (Flash2000, Thermo Fisher Scientific) was employed. The objective of this analysis was to monitor the decline in the C content as a result of the chemical treatments. A field emission scanning electron microscope (FE-SEM, 7800F Prime, JEOL), X-ray photoelectron spectroscopy (XPS, NEXSA, Thermo Fisher Scientific), and Fourier transform infrared spectrometer (FT-IR, TENSOR27, Bruker) were employed to support the successful carbonization and activation. The adsorption performance was determined using a UV-vis spectrometer (UV-2450, Shimadzu).

The adsorption performance of the samples under various initial dosages (1–3 g) using 50 mg L^−1^ of toluene and benzene in a 1 L beaker at 25 °C was determined. Additionally, the former using various temperature ranges (25–45 °C) was determined using 50 mg L^−1^ of VOCs in a 1 L beaker with 3 g of absorbents. The further conditions in adsorption studies are listed at the beginning of each study. In all cases, the experimental environment was sealed using laboratory wrapping film to minimize the evaporation of VOCs in the aqueous solution. The calculation methods for the adsorption phenomenon and regeneration method are also described in the subsequent paragraphs. Lastly, the chemical and physical properties of the selected VOCs are listed in Table 1.

## 3. Results and Discussion

**Figure 1:**
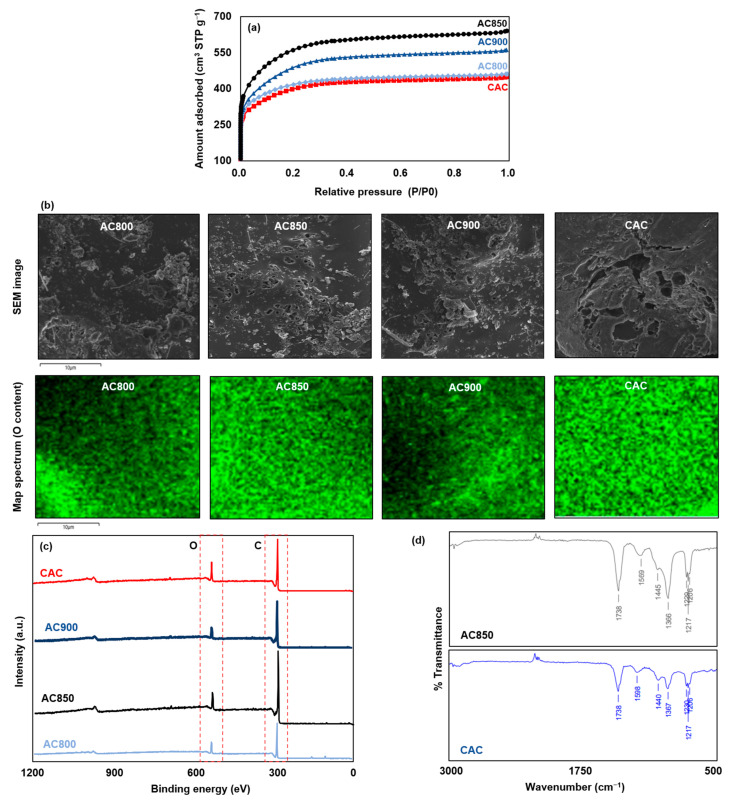
The N_2_ adsorption–desorption characteristics of samples are shown in (**a**). SEM and the following O content is mapped in (**b**). XPS and FT-IR results of samples are shown in (**c**,**d**), respectively.

Figure 1 and Table 2 display the surface characteristics of the samples. The BET surface area of the AC samples is in the range of 622–1047 m^2^ g^−1^. The activation temperature is one of the critical factors that influence the pore characteristics. A low activation temperature may limit the development of micropores. On the other hand, a high activation temperature may collapse the pore structure due to the thermal decomposition of the carbon framework. In this work, 850 °C seems to be the optimal activation temperature with the highest BET surface among the AC samples. Thus, we abbreviate AC850 as AC sample and compared it with the CAC sample in the toluene and benzene adsorption. The amount of micropores (<2 nm) plays a vital role in the selectivity and kinetics of the adsorption process [18]. The CAC sample shows the highest micropore ratio of 30.33%, showing that the activation was effectively complete. However, the CAC sample revealed the lowest BET surface due to the growth of the surface functional groups. The activation process can lead to the formation of new surface functional groups that may be less favorable for gas adsorption or may occupy some of the available adsorption sites. For example, carbonyl (-C=O) groups with heat can cause them to be converted to carboxyl (-COOH) groups, which can be less favorable for gas adsorption. Additionally, the formation of new functional groups may decrease the available surface area for gas adsorption by occupying some of the active sites. All the samples were dominated by the C content with some O content, which is attributed to the surface functional groups during the activation processes.

**Figure 2:**
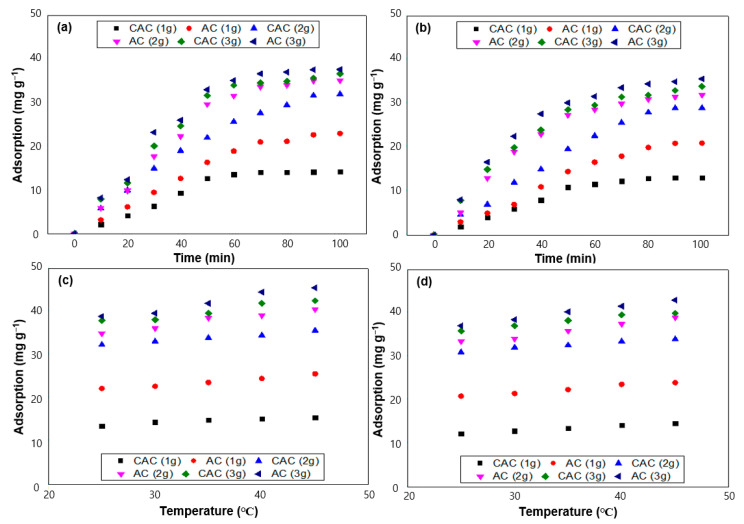
Adsorption performance of samples by various initial dosage is listed in (**a**) toluene and (**b**) benzene. The former with fixed dosage (3 g) of samples by various temperatures is shown in (**c**) toluene and (**d**) benzene.

The results obtained from the XPS and FT-IR spectroscopy confirmed the trend mentioned above. XPS is a technique that focuses on the surface of the material and can provide information regarding the composition of the elements present and their chemical bonding. In general, the XPS spectra for the AC sample and CAC sample were found to be similar. The O peak observed in both samples is attributed to surface functional groups such as carbonyl and carboxyl. Typically, the O peak appears within the range of 550–600 eV, with the O1s peak appearing in the range of 530–540 eV. The C peak is generally associated with the content of carbon in the aromatic and aliphatic structures, with the C1s peak appearing within the range of 280–300 eV. All the samples displayed distinct O and C peaks. The intensity of the O and C peaks were found to be inversely correlated due to the presence of surface functional groups. The peaks observed in the FT-IR spectrum correspond to the vibrational frequencies of various bonds in the molecule, and the intensity and position of these peaks can provide significant information regarding the chemical structure of the molecule. The types of functional groups typically associated with the vibrational frequencies observed in Figure 2d are listed in Table 3. The activation process resulted in both samples displaying a significant spectrum of surface functional groups. The XPS and FT-IR results successfully supported the aforementioned observations.

Before commencing the adsorption study, it is imperative to acknowledge that the evaporation rate of toluene and benzene in water may introduce an error in the analysis. The estimation of the evaporation rate of these compounds, as well as that of water, can be conducted using the formula provided in Equation (1).

Evaporation rate = K × A × (P − Pw)(1)

Herein, K represents the mass transfer coefficient in m s^−1^, A is the surface area of the liquid exposed to air in m^2^, P denotes the vapor pressure of the compound in Pa, and Pw indicates the vapor pressure of water in Pa. Assuming a mass transfer coefficient of 0.1 cm s^−1^, a surface area of approximately 0.016 m^2^ for the 1 L beaker, and vapor pressures of 30 kPa, 12.5 kPa, and 3.2 kPa for toluene, benzene, and water at 25 °C, respectively. The estimated evaporation rates for toluene and benzene are 1.2 mg min^−1^ and 0.5 mg min^−1^, respectively, while that of water is 0.2 mg min^−1^. However, it is worth noting that, in a closed environment where the beaker is sealed with laboratory wrapping film, the estimated evaporation rates of toluene and benzene in a 1 L beaker of water with an initial concentration of 50 mg L^−1^ are 0.0022 mg min^−1^ and 0.0009 mg min^−1^, respectively. Therefore, the total evaporation (experimental error) of the selected VOCs in the moderate temperature is expected to be at least 22 mg and 9 mg for toluene and benzene at 100 min, respectively.

To explicate the adsorption performance of toluene and benzene onto two distinct samples, 1–3 g of each sample were introduced, with 50 mg L^−1^ of the selected VOCs. The thermal environment was purposefully manipulated over a range from 25 to 45 °C. As discernible from Figure 2a,b, the superior surface characteristics of the AC sample engender a greater number of sites available for the adsorption of VOCs, thereby resulting in superior adsorption performance relative to the CAC sample. Correspondingly, a positive correlation between the initial sample quantity and the adsorption performance of toluene and benzene is evident from the observed increase in the adsorption of VOCs in Figure 2c,d. This phenomenon can be attributed to several underlying causes. Firstly, the heightened diffusion, as the rate of toluene and benzene molecule diffusion into the samples governs their adsorption performance. At elevated temperatures, the kinetic energy of the VOC molecules rises, promoting their entering into the pores of the samples, which, correspondingly, facilitates faster adsorption. Secondly, the increased surface activity owing to the greater abundance of oxygen functional groups at elevated temperatures renders the samples more reactive, thereby augmenting the available sites for the adsorption of VOCs, as evidenced in Figure 1d where both samples possess a considerable proportion of oxygen functional groups. Lastly, the reduced intermolecular forces between the VOC molecules and the samples, as a result of the increasing temperature, lower the energy barrier for the adsorption process and consequently facilitate the adsorption of VOCs onto the samples.

**Figure 3:**
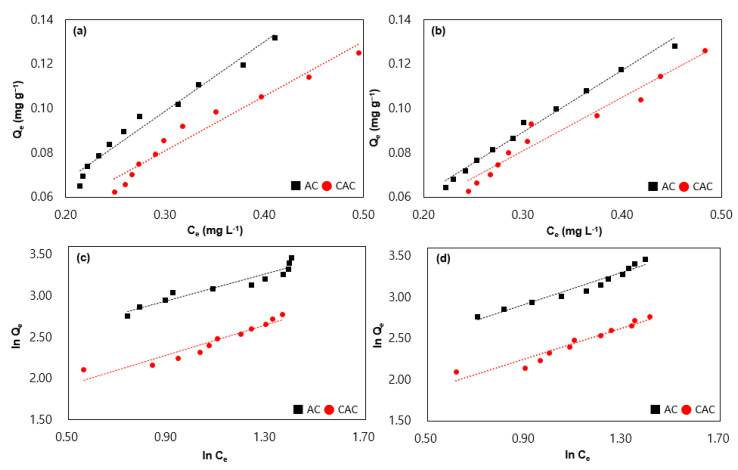
Langmuir isotherm of samples by various concentrations of toluene and benzene is depicted in (**a**,**b**), while Freundlich isotherm of the former (**c**,**d**).

By fitting experimental data to a particular isotherm model, one can obtain parameters that characterize the adsorption properties of an adsorbent, such as the maximum adsorption capacity and the affinity of the adsorbent for the adsorbate. Here, the Langmuir and Freundlich isotherms were employed to discern the adsorption trends as depicted in Figure 3 and Table 4 [19,20,21]. The experimental setup was performed by using 3 g of the samples with 10–20 mg L^−1^ of the selected VOCs. The Langmuir isotherm is a model that elucidates the adsorption of a solute onto a solid surface. This model postulates a monolayer of adsorbate molecules on the adsorbent surface, with adsorption occurring solely at specific sites on the surface. The Langmuir isotherm equation is expressed as Equation (2).

Q = Q_m_K_L_C_e_/(1 + K_L_C_e_) (2)

where Q represents the amount of adsorbate adsorbed per unit mass of adsorbent, C_e_ is the concentration of adsorbate in the solution, K_L_ is the Langmuir constant, and Q_m_ denotes the maximum adsorption capacity. A greater Q_m_ value corresponds to an increased adsorption capacity, while a lower K_L_ value is indicative of a greater affinity for the adsorbate. From the presented Langmuir isotherm data, it is apparent that the AC sample (196.0784 mg g^−1^) exhibits a greater Q_m_ value compared to the CAC sample (135.1351 mg g^−1^) for toluene. The benzene adsorption showed a similar trend of AC (181.8182 mg g^−1^) > CAC (116.2791 mg g^−1^). This observation suggests that the AC sample contains a greater number of adsorption sites, hence meaning it is capable of adsorbing a higher amount of toluene and benzene per unit mass of adsorbent than the CAC sample. Furthermore, the K_L_ value for the AC sample is lower than that of the CAC sample, implying that the AC sample exhibits a greater affinity for the selected VOCs.

The Freundlich isotherm represents an established model employed to explicate the process of adsorption of solutes onto a solid surface. The aforementioned model postulates the occurrence of adsorption on numerous layers of the adsorbent surface, while stipulating a decline in the adsorption energy corresponding to an increase in the number of adsorbed molecules. The equation denoting the Freundlich isotherm, delineated as Equation (3), is as follows:

Q = K_f_ C_e_^1/n^ (3)

where K_f_ corresponds to the Freundlich constant, and 1/n measures the adsorption intensity. An enhanced K_f_ value implies a heightened adsorption capacity, whereas a diminished 1/n value signifies increased adsorption intensity. Based on the data pertaining to the Freundlich isotherm, the sample of AC surpasses the CAC sample in terms of its K_f_ value. This disparity suggests a superior adsorption capacity of the AC sample in relation to the selected VOCs when compared to the CAC sample. Furthermore, the 1/n value of the AC sample appears to be lower than that of the CAC sample, signifying an augmented adsorption intensity for the selected VOCs in the case of the AC sample. The correlation coefficient is a statistical measure of how well the experimental data fit the model. The closer the R^2^ value is to 1, the better the model fits the data. The R^2^ value of Langmuir isotherm (avg. 0.9669) is higher than the Freundlich isotherm (avg. 0.9298) in all cases, implying that the adsorption process follows a monolayer adsorption mechanism.

The utilization of the pseudo-first-order and pseudo-second-order kinetics has been employed to explicate the adsorption behavior [22,23,24,25]. The pseudo-first-order kinetic model postulates that the adsorption rate is directly proportional to the dissimilarity in the concentration between the solution and the adsorbent surface. The model can be mathematically represented as Equation (4), where Q_e_ signifies the quantity of VOCs adsorbed at equilibrium, Q_t_ denotes the quantity of VOCs adsorbed at time t, K_1_ denotes the rate constant of the adsorption process, and R^2^ indicates the correlation coefficient.

log(Q_e_ − Q_t_) = log(Q_e_) − K_1_t/2.303 (4)

Conversely, the pseudo-second-order kinetic model assumes that the adsorption rate is directly proportional to the square of the concentration difference between the solution and the adsorbent surface. The model is expressed as Equation (5), where K_2_ denotes the rate constant of the adsorption process.

t/Q_t_ = 1/K_2_Q_e_^2^ + t/Q_e_ (5)

**Figure 4:**
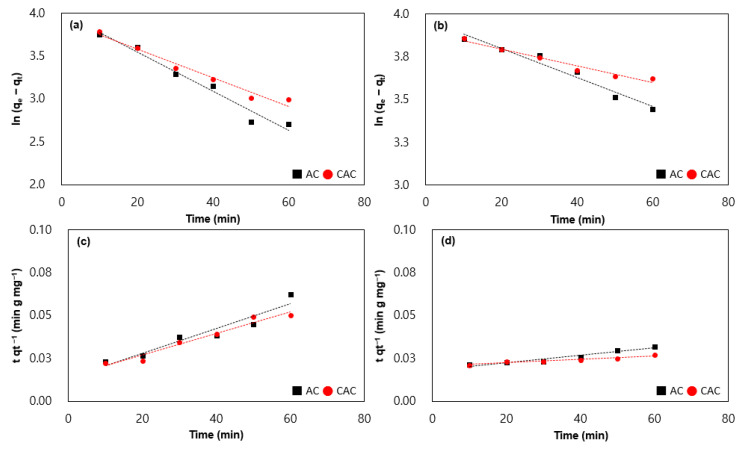
Pseudo-first-order kinetics of samples are depicted in (**a**) toluene and (**b**) benzene, while pseudo-second-order of the former (**c**,**d**).

The present study employed 3 g of adsorbents and 50 mg L^−1^ of VOCs at various time intervals ranging from 10 to 60 min (Figure 4 and Table 5). The Q_e_ values obtained from the two models demonstrate that the AC sample exhibited better adsorption performance in comparison to the CAC sample. The values of K_1_ obtained from the pseudo-first-order kinetic model for both activated carbon samples ranged from 0.0049 to 0.2290, signifying a relatively sluggish adsorption process. Furthermore, the R^2^ values obtained from the model were high (>0.95), indicating a good fit of the experimental data to the model. The values of K_2_ obtained from the pseudo-second-order kinetic model for both samples ranged from 0.0001 to 0.0007, indicating a relatively swift adsorption process. However, the R^2^ values obtained from the model were slightly lower than those obtained from the pseudo-first-order kinetic model, signifying a relatively inferior fit of the experimental data to the model. Notably, the AC sample, which possessed a higher surface area, exhibited superior adsorption performance than the CAC sample. This is in concordance with the fact that the surface area of the adsorbent is a crucial determinant affecting the adsorption behavior of the VOCs onto adsorbents. In all cases, the pseudo-first-order kinetic model provided a better fit to the experimental data compared to the pseudo-second-order kinetic model. In other words, the rate of adsorption is directly proportional to the concentration difference between the solution and the sample surface.

The Gibbs energy change (∆G_ads), enthalpy change (∆H_ads), and entropy change (∆S_ads) are key thermodynamic properties that determine the direction and stability of the chemical reactions, including adsorption. The adsorption process can be assessed by analyzing these thermodynamic parameters. The Gibbs–Helmholtz equation can be used to determine the trend of adsorption (Equation (6)).

∆G_ads = ∆H_ads − T∆S_ads(6)

where T is the temperature in Kelvin. The experimental data, such as the adsorption isotherms, can be used to calculate ∆G_ads. The Gibbs adsorption isotherm relates the amount of adsorbate to the equilibrium constant, K, which is related to the Gibbs energy of the adsorption (Equation (7)).

∆G_ads = −RT ln(K)(7)

where R is the gas constant. In this study, the adsorption of 50 mg L^−1^ of VOCs by 3 g of adsorbents was analyzed at varying temperature intervals ranging from 25 to 45 °C (Table 6). The obtained ∆G_ads values for both VOCs decreased with the increasing temperature, indicating that the adsorption process is spontaneous and favorable. This suggests that the adsorbate molecules are attracted to the adsorbent surface and are likely to be adsorbed onto it as the adsorption process releases energy to the system. The positive ∆H_ads values indicate that the process is endothermic. A positive value of ∆S_ads would imply an increase in the system’s disorder or randomness during the adsorption process. This spontaneous trend is commonly observed in adsorption using carbonaceous absorbents [26,27,28,29].

**Figure 5:**
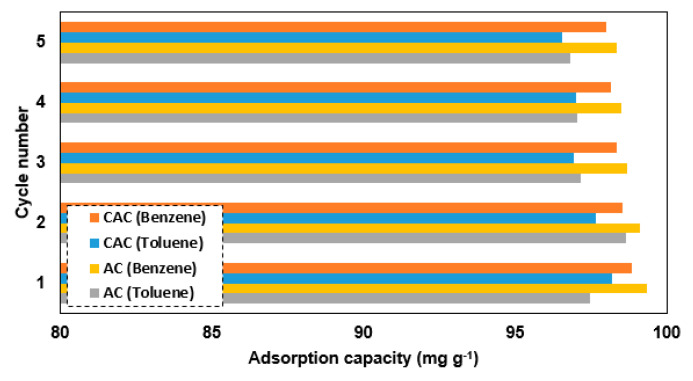
Regeneration efficiency of samples (3 g) with 50 mg L^−1^ of selected VOCs.

**Figure 6:**
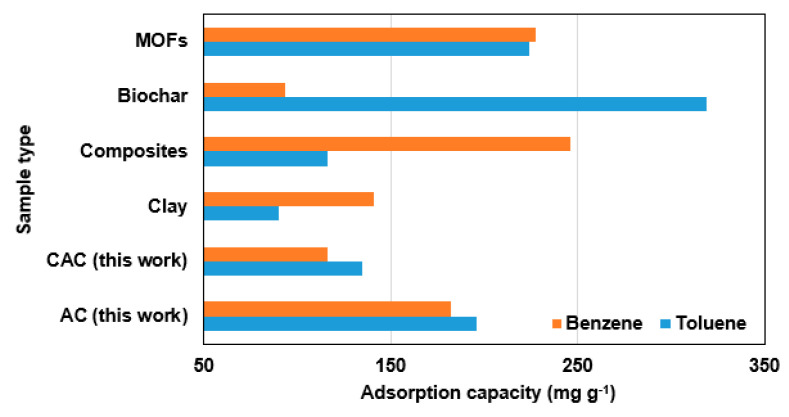
Adsorption capacity of selected VOCs by various sample types.

One can observe the desorption efficiency of carbonaceous adsorbents for VOCs through the application of heat. The thermal energy provided by higher temperatures allows for the detachment of VOCs from the surface of samples, thus improving the desorption efficiency. Previous investigations have demonstrated that temperatures ranging from 200–300 °C can achieve desorption efficiencies of up to 95% for VOCs [30,31]. In this study, the desorption of samples was performed under an N_2_ atmosphere as the temperature was gradually increased to 300 °C at a rate of 10 °C min^−1^. Afterward, the weight of the heated sample was measured and utilized as the initial quantity for the subsequent adsorption cycle. Figure 5 illustrates that all the samples exhibited a consistent cycle of regeneration efficiency after undergoing five cycles, thus indicating a reliable performance. Here, benzene showed a better regeneration performance compared to toluene. The adsorption capacity of the samples in comparison to other works is shown in Figure 6 [12]. Palygorskite clay and ZIF-67 MOF can adsorb more benzene than the AC sample because of their unique porous structure and coordinatively unsaturated metal sites, respectively. Meanwhile, the diatomite/silicalite-1 composite has a higher toluene adsorption capacity than the AC sample because of its special hierarchical pore structure. The zeolite/GO composite has a higher benzene adsorption capacity than the AC sample due to its large surface area and oxygen functional groups. On the other hand, biochar created from cotton stalks and date palm pits have lower adsorption capacities for both VOCs than the AC sample, except for the date palm pits’ higher adsorption capacity for toluene due to its higher concentration of alkaline earth metals. Compared to other adsorbents, the CAC sample has a lower adsorption capacity for both VOCs than palygorskite clay, ZIF-67, and zeolite/GO, but is higher than cotton stalk biochar for toluene and montmorillonite clay for benzene. Both samples are promising due to their simple synthesis and cost efficiency, but their adsorption capacity can be improved through chemical activation or functionalization.

## 4. Conclusions

This study explores the adsorption phenomenon of toluene and benzene onto two types of activated carbons. The AC and CAC samples exhibited a reasonably high BET surface area of 1047 m^2^ g^−1^ and 610 m^2^ g^−1^, respectively. The activation process introduced surface functional groups, allowing for the effective adsorption of VOCs. To analyze the adsorption trend of the samples, we utilized the Langmuir and Freundlich isotherms. The adsorption capacity of toluene on the AC sample was 196.0784 mg g^−1^, whereas the CAC sample adsorbed 135.1351 mg g^−1^. Similarly, the adsorption capacity of benzene onto the AC sample was 181.8182 mg g^−1^, and the CAC sample adsorbed 116.2791 mg g^−1^. The findings indicate that the AC sample with a high surface area exhibited superior adsorption performance compared to the CAC sample. Our results further suggest that the adsorption process follows a monolayer adsorption mechanism, as indicated by the higher coefficient of the Langmuir isotherm (avg. of R^2^ = 0.9669) compared to the Freundlich isotherm (avg. of R^2^ = 0.9298). Additionally, the pseudo-first-order kinetic model (avg. of R^2^ = 0.9654) was found to be more fitting than the pseudo-second-order kinetic model (avg. of R^2^ = 0.9297), indicating that the rate of adsorption is directly proportional to the concentration difference between the solution and the sample surface. Through our calculations of ∆G_ads, we determined that the observed adsorption trend was favorable and spontaneous. The positive values of both ∆H_ads and ∆S_ads indicate an endothermic and disordered system environment, respectively. Furthermore, we found that all samples recorded more than 95% of regeneration efficiency at the fifth round, thus highlighting the reusability of the activated carbon materials. It is crucial to acknowledge that the experimental data obtained in this study were collected under specific conditions and may not be generalizable to all industrial settings. Therefore, future studies should focus on exploring the chemically modified samples and their adsorption performance on VOCs. By examining the various surface chemistries of samples and their concurrent relationships to VOCs adsorption, we can better understand the intricate mechanisms of adsorption and improve the effectiveness of VOC removal from various environments.

## Figures and Tables

**Table 1:** The main properties of toluene and benzene.

VOCs	Molecular Formula	Molecular Weight (g/mol)	Boiling Point (°C)	Melting Point (°C)	Dipole Moment (D)	Refractive Index	Kinetic Diameter (nm)
Toluene	C_7_H_8_	92.14	110	−95	0.45	1.49	0.68
Benzene	C_6_H_6_	78.11	80.1	5.5	non-polar	1.501	0.67

**Table 2:** The physical and chemical surface of samples.

Sample	BET (m^2^ g^−1^)	Micropore (m^2^ g^−1^)	Macropore (m^2^ g^−1^)	C Content (%)	O Content (%)
Raw-pitch	21.1	-	-	-	-
AC800	896	622	274	92.46	1.09
AC850	1047	910	137	87.10	2.01
AC900	951	700	251	91.92	0.96
CAC	610	490	75	84.75	2.25

**Table 3:** Visible FR-IR spectra of samples.

Functional Group	Wavenumber (cm^−1^)
Carbonyl	1730–1740
Benzene	1590–1600
Methyl	1440–1450
Methylene	1360–1370
Amine	1200–1220

**Table 4:** Langmuir and Freundlich constants of samples by various concentrations of toluene and benzene.

**Isotherm**	**VOC**	**Sample**	**Constant**		**R^2^**
			**Q_m_ (mg g^−1^)**	**K_L_ (1 mg^−1^)**	
Langmuir	Toluene	AC	196.0784	0.0163	0.9705
		CAC	135.1351	0.3017	0.9556
	Benzene	AC	181.8182	0.0197	0.9892
		CAC	116.2791	0.0357	0.9523
**Isotherm**	**VOC**	**Sample**	**Constant**		**R^2^**
			**K_f_ (mg g^−1^)**	**1/n**	
Freundlich	Toluene	AC	9.1276	1.0235	0.9212
		CAC	4.3545	1.0994	0.9142
	Benzene	AC	7.7408	1.0338	0.9497
		CAC	4.0980	1.0638	0.9340

**Table 5:** Pseudo-first-order and pseudo-second-order kinetic parameters of samples.

**Kinetic**	**VOC**	**Sample**	**Constant**		**R^2^**
			**Q_e_**	**K_1_**	
Pseudo-first-order	Toluene	AC	55.1083	0.2290	0.9673
		CAC	50.0339	0.0120	0.9691
	Benzene	AC	52.9369	0.0085	0.9615
		CAC	49.0284	0.0049	0.9635
**Kinetic**	**VOC**	**Sample**	**Constant**		**R^2^**
			**Q_e_**	**K_1_**	
Pseudo-second-order	Toluene	AC	72.4638	0.0007	0.9146
		CAC	68.9655	0.0006	0.9575
	Benzene	AC	55.5556	0.0002	0.9452
		CAC	49.0196	0.0001	0.9015

**Table 6:** Thermodynamic parameters for VOCs adsorption on the samples by various temperature ranges.

**Sample**	**Target**	**Temperature (°C)**	**∆H_ads** **(kJ mol^−1^ K)**	**∆S_ads** **(kJ mol^−1^ K)**	**∆G_ads** **(kJ mol^−1^ K)**
AC	Toluene	25	2.1565	35.1017	−8.3293
		35			−9.0632
		45			−9.7306
CAC		25	1.5830	33.1421	−8.3143
		35			−9.0171
		45			−9.6365
**Sample**	**Target**	**Temperature (°C)**	**∆H_ads**	**∆S_ads**	**∆G_ads**
AC	Benzene	25	1.7337	33.6384	−8.3215
		35			−9.0027
		45			−9.6663
CAC		25	1.5289	32.9151	−8.3000
		35			−9.0001
		45			−9.6125

## Data Availability

Applicable by request.
